# A significant correlation between C - reactive protein levels in blood monocytes derived macrophages versus content in carotid atherosclerotic lesions

**DOI:** 10.1186/1476-9255-11-7

**Published:** 2014-03-03

**Authors:** Marielle Kaplan, Shadi Hamoud, Yevgeny Tendler, Edna Meilin, Aviva Lazarovitch, Samy Nitecki, Tony Hayek

**Affiliations:** 1 Laboratory of Clinical Biochemistry, Rambam Health Care Campus, Haifa, Israel; 2 Department of Internal Medicine E, Rambam Health Care Campus, Haifa, Israel; 3 Lipid Research Laboratory, The Ruth and Bruce Rappaport Faculty of Medicine, Technion, Haifa, Israel; 4 Department of Vascular Surgery, Rambam Health Care Campus, Haifa, Israel

**Keywords:** C-reactive protein, Atherosclerosis, Macrophages, Inflammation

## Abstract

**Background:**

Atherosclerosis is a complex disease involving different cell types, including macrophages that play a major role in the inflammatory events occurring in atherogenesis. C-Reactive Protein (CRP) is a sensitive systemic marker of inflammation and was identified as a biomarker of cardiovascular diseases. Histological studies demonstrate CRP presence in human atherosclerotic lesions, and we have previously shown that macrophages express CRP mRNA. CRP could be locally secreted in the atherosclerotic lesion by arterial macrophages and local regulation of CRP could affect its pro-atherogenic effects. Moreover, human blood derived macrophages (HMDM) expression of CRP could reflect atherosclerotic lesion secretion of CRP.

**Methods:**

Ten type 2 diabetic patients and ten non-diabetic patients scheduled to undergo carotid endarterectomy were enrolled in this study, and their blood samples were used for serum CRP, lipid determination, and for preparation of HMDM further analyzed for their CRP mRNA expression and CRP content. Carotid lesions obtained from the patients were analyzed for their CRP and interleukin 6 (IL-6) content by immunohistochemistry.

**Results:**

Lesions from diabetic patients showed substantially higher CRP levels by 62% (p = 0.05) than lesions from non diabetic patients, and CRP staining that co-localized with arterial macrophages. CRP carotid lesion levels positively correlated with CRP mRNA expression (r^2^ = 0.661) and with CRP content (r^2^ = 0.611) in the patient’s HMDM.

**Conclusions:**

Diabetes up-regulated carotid plaques CRP levels and CRP measurements in HMDM could reflect atherosclerotic lesion macrophages secretion of CRP. Understanding the regulation of locally produced macrophage CRP in the arterial wall during atherogenesis could be of major importance in identifying the underlying mechanisms of inflammatory response pathways during atherogenesis.

## Introduction

Arterial inflammation represents a key feature determining the course of atherogenesis. At early stages of atherogenesis, blood monocytes derived macrophages play a major role in the inflammatory events occurring in the atherosclerotic lesion. During atherogenesis, monocytes derived macrophages transform into foam cells, thus causing an amplification of the local inflammatory activity in the arterial wall, resulting in atherosclerotic plaque destabilization and vulnerability [1-3]. Diabetes mellitus in general and hyperglycemia in particular are associated with premature and accelerated atherosclerosis, mediated by induction of pro-inflammatory processes [4].

C-Reactive Protein (CRP) is a sensitive systemic marker of inflammation and was first identified as a biomarker of cardiovascular diseases [5-9]. The issue of whether CRP is a critical, active component of the inflammatory cascade during atherosclerosis or simply a marker of inflammation remains controversial [10,11]. CRP is primarily synthesized by hepatocytes triggered by the dual activity of interleukin 6 (IL-6) and IL-1 [12], and participates to the enhancement of the inflammatory cascade by inducing IL-6 secretion [13]. However recently, several studies discovered CRP in coronary and carotid atheroma [14,15]. These observations have resulted in an ongoing debate on the potential source of CRP within the atherosclerotic tissue. Several studies, including our recent study, have identified various cell-types that may serve as extrahepatic sources for CRP-production, including macrophages [16,17].

In addition, recent histological investigations have demonstrated the presence of CRP in the human arterial intima of atherosclerotic lesions. Detection of both CRP mRNA and protein within atherosclerotic lesions, predominantly localized to vascular smooth muscle cells and macrophages, argues in favor of *de novo* synthesis in the vessel wall, disputing the idea of lesional CRP that derives only from deposition of the systemic circulation [18,19].

We hypothesized that CRP could be locally secreted in the atherosclerotic lesion by arterial macrophages and that pro-atherosclerotic triggers such as diabetes could up-regulate atherosclerotic lesion secretion of CRP. Moreover, the expression of CRP in blood derived macrophages could reflect atherosclerotic lesion secretion of CRP.

Our aims were to evaluate whether diabetes increases CRP expression in arterial macrophages and to study a possible correlation between CRP expression in human monocytes derived macrophages (HMDM) and CRP content in carotid atherosclerotic plaques.

## Materials and methods

### Patients

Twenty Patients (10 type 2 diabetic patients and 10 non-diabetic patients) that were scheduled to undergo Carotid Endarterectomy were enrolled in this study. All patients sign an informed consent and the study was approved under Helsinky committee approval #0247-09 (Rambam Health Care Campus Internal Helsinky committee).

Blood samples isolated from the patients were used for serum CRP and lipid determination, as well as for preparation of human monocytes derived macrophages (HMDM). Carotid lesions obtained from the patients were analyzed for their CRP content and macrophages localization by immunohistochemistry.

### Serum determinations

Serum cholesterol, triglycerides, HDL-Cholesterol and glucose were determined by commercial kits (Siemens, Germany) using an autoanalyzer dedicated instrument (Dimension RXL, Siemens, Germany). High-sensitivity CRP levels were measured with latex-enhanced immunonephelometry on a Siemens BN-ProSpec Nephelometer (Siemens, Germany). Interleukin 6 levels were determined using an ELISA assay (R&D systems, MN, USA).

Hemoglobin A1C was measured by HPLC using the BIORAD D-10 instrument (BIORAD, CA, USA).

### Human monocyte derived macrophages (HMDM) isolation

Human monocyte-derived macrophages (HMDM) were prepared from the blood of fasting diabetic and non-diabetic patients by density gradient centrifugation [20]. Twenty milliliters of blood anticoagulated with sodium heparin (final concentration, 10 U/ml) were layered over 15 ml Ficoll-Paque. After centrifugation at 500×g for 30 minutes at 23°C, the mixed mononuclear cell band was removed by aspiration and the cells were washed twice at 4°C in RPMI-1640 supplemented with 2 mM glutamine, 1 mM pyruvate, 100U/ml penicillin, 100 μg/ml streptomycin. The cells were plated at 3×105 monocytes per 16-mm dish (Primaria Brand, Falcon Labware) in the same medium (0.5 ml) containing 20% autologous serum (cells are incubated with the associated patient’ serum). After two hours of incubation at 37°C in 5% CO2, 95% air, non-adherent cells were removed by three washes with serum-free medium. The cells were placed in fresh medium containing 20% autologous serum thus enabling us to imitate the cells in vivo surrounding, fed twice weekly, and used for experiments after seven days in culture.

### Quantitative CRP mRNA determination by real time PCR

RNA was extracted from tissue or cells using MasterPure™ RNA purification kit (Epicentre Biotechnologies, Madison, WI, USA). cDNA was prepared using Verso™ cDNA kit (Thermo Scientific, Epsom, UK). Primers and probes for β-actin, and human CRP genes were designed by Primer Design, Southampton, UK. Using ABsolute Blue QPCR ROX mix (Thermo Scientific), expression was determined by quantitative real-time PCR with Rotor-Gene 6000 amplification detection system.

### Carotid lesions determination of CRP and IL-6 by immunohistochemistry

Carotid artery samples were collected immediately, frozen in liquid nitrogen and stored in -75°C. Air-dried serial frozen sections were fixed in ice-cold acetone and exposed to the following primary antibodies: monoclonal mouse anti-human CD68, clone PG-M1 (1:100, Santa Cruz Biotechnology, Inc., Santa Cruz, USA), goat anti-human IL-6 (1:100, R & D Systems, Inc, USA) and monoclonal mouse Anti-C-Reactive Protein, clone CRP-8 (1:100, Sigma-Aldrich, USA), The immuno-reactions were visualized using the DAKO LSAB method and DAKO LSAB + Kit, HRP (DAKO, Denmark) [21]. Controls without primary antibodies were run for each protocol, resulting in consistently negative results. Isotype controls were tested with normal serum from the same animal or species as the primary antibody or the same immunoglobulin isotype. The slides were washed, mounted with an aqueous mounting medium and photographed within a few hours under a digital microscope camera (Olympus Upright Light Microscope). Immunohistochemical quantification for CRP and IL6 were assessed by image processing software “Image Pro Plus version 6”. Light intensity and contrast were standardized for a respective sections with an appropriate control. For analysis of CRP and IL6 staining three 200 μM × 200 μM areas where chosen by blinded analysis from each region and reported as an average of area IOD.

### Cellular determination of CRP and IL-6 by immunohistochemistry

Human monocytes derived macrophages (HMDM) plated on a round caver glass at 16-mm dish were fixed in the absolute methanol for 10 min. After rinsing with cold PBS (pH 7.4) cells were permeabilized with 0.5% Triton X-100 for 10 minutes at room temperature. After blocking, antihuman-CRP antibody clone CRP-8 (Sigma-Aldrich, USA) and goat anti-human IL-6 (1:00, R & D Systems, Inc, USA) were respectively added and incubated at room temperature for 2 hours followed by incubation with CyTM3 conjugated Donkey Anti-mouse IgG (Jackson Immuno Research Labs, Baltimore, USA) and FITC conjugated Rabbit Anti-Goat IgG (Jackson Immuno Research Labs, Baltimore, USA) for 1 hour. Following removal of the antibodies, and nucleus counterstaining with To-pro-3 iodide (Invitrogen, Carlsbad, Ca, USA). Cells were rinsed with PBS, cover glass with cells removed from dish and mounted with UltraMount (Lab Vision, UK) [22]. All control samples were processed without primary antibody. Fluorescence was immediately observed using either an Axioscop 2 or Leica laser scanning confocal microscope (Bensheim, Germany) with image processing software “Image Pro Plus version 6”. Light intensity and contrast were standardized for respective cells samples with an appropriate control.

### Statistics

Analysis of variance (ANOVA) was used for all statistical analyses. Results are expressed as mean ± SD.

## Results

Ten diabetic patients (HbA1C =7.7 ± 1.4%, fasting serum glucose of 156 ± 41 mg/dl) and ten non diabetic patients (with fasting serum glucose 102 ± 12 mg/dl) that were scheduled to undergo endarterectomy, were enrolled in the study (Table 1). There were no significant differences regarding the male and female distribution as well as the mean age between diabetic patients versus non-diabetic patients (Table 1). Both groups of patients exhibit similar concomitant diseases such as hypertension, ischemic heart diseases hypertension and hyperlipidemia (Table 1). Moreover, there were no significant differences in drugs treatment (besides hypoglycemic therapy) between diabetic patients versus non-diabetic patients (Table 1).

**Table 1:** Characteristics of diabetic patients versus non-diabetic patients, including serum lipid profile (Total cholesterol, LDL-Cholesterol, Triglycerides and HDL-Cholesterol levels), serum CRP levels, concomitant disease and drug therapy

	**Diabetic patients (n = 10)**	**Non-diabetic patients (n = 10)**	**P value**
Total Cholesterol (mg/dl)	163 ± 25	176 ± 30	N.S.
LDL (mg/dl)	94 ± 27	99 ± 26	N.S.
Triglycerides (mg/dl)	141 ± 40	142 ± 90	N.S.
HDL (mg/dl)	41 ± 10	50 ± 12	N.S.
hCRP (mg/dl)	5.2 ± 3.7	2.8 ± 1.8	0.1
Hemoglobin A1c level (%)	7.73		
Mean Age (years)	67.3	67.7	N.S.
Females (%)	40	50	N.S.
Males (%)	60	50	N.S.
Hypertension (%)	90	90	N.S.
Hyperlipidemia (%)	100	80	N.S.
Smoking history (%)	60	45	N.S.
Ischemic heart disease (%)	60	60	N.S.
History of cerebrovascular disease (%)	50	40	N.S.
Known PVD (%)	30	20	N.S.
Aspirin/clopidogrel (% treated)	90	100	N.S.
Warfarin (% treated)	20	20	N.S.
Beta Blockers (% treated)	70	80	N.S.
Calcium Channel Blockers (% treated)	50	50	N.S.
Angiotensin receptors blockers/ACE-inhibitors (% treated)	50	70	N.S.
Statins (% treated)	100	90	N.S.
Hypoglycemic drugs (% treated)	90	0	<0.01
Symptomatic and asymptomatic patients (%)	30% symptomatic	30% symptomatic	N.S.
	70% asymptomatic	70% asymptomatic	
Time elapsed from diagnosis leading to endaterectomy (weeks)	5.9 ± 6.7	6.6 ± 7.8	N.S.

(N.S.: non-significant).

The blood lipid profile was similar in both diabetic and non diabetic patients as measured by their total cholesterol, LDL-Cholesterol, triglycerides and HDL-Cholesterol (Table 1). The mean CRP level was higher by 1.9 fold in the diabetic patients group than in the control group but this increment was without statistical significance (p = 0.1, Table 1).

**Figure 1:**
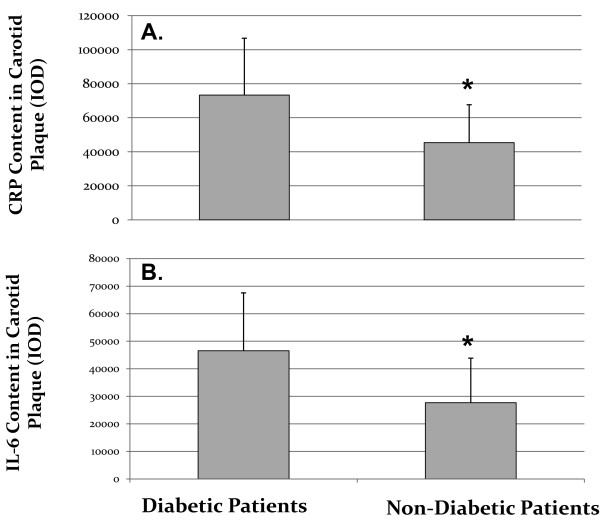
**Carotid lesions samples obtained from diabetic and non-diabetic patients were analyzed for their C-Reactive Protein (CRP) content (A) and Interleukin-6 (IL-6) content (B) by immunohistochemistry.** * = p < 0.05, n = 10 samples for each group of patients; (IOD: Integrated optical density).

**Figure 2:**
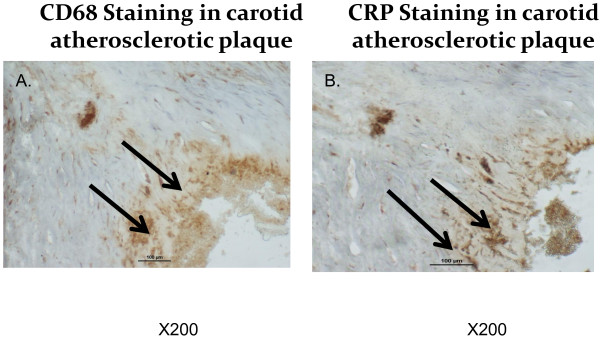
**Representative photomicrographs of carotid lesions samples obtained from diabetic and non-diabetic patients that were stained with CD68- a marker of human macrophages (A), as well as for C-Reactive Protein (CRP) content (B).** Arrows indicate positive staining location. Representative from n = 10 samples for each group of patients.

**Figure 3:**
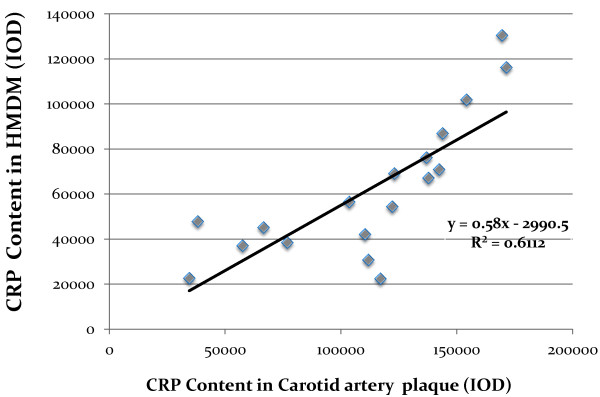
**Correlation between C-Reactive Protein (CRP) levels measured in Human Monocytes derived macrophages (HMDM) and CRP levels measured in the carotid lesion determined by immunohistochemistry, from both diabetic and non-diabetic patients.***R*^*2*^*: Coefficient of correlation; (IOD: Integrated optical density).*

**Figure 4:**
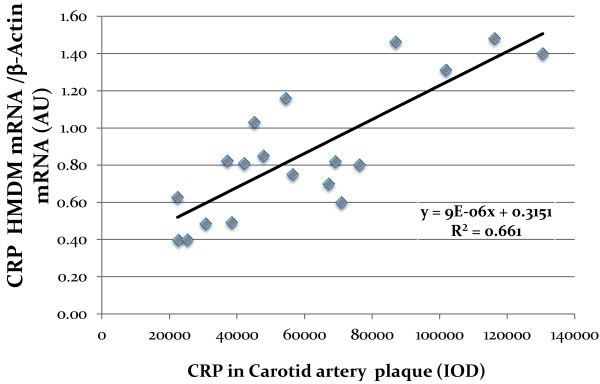
**Correlation between C-Reactive Protein (CRP) mRNA levels measured in Human Monocytes derived macrophages (HMDM) and CRP levels measured in the carotid lesion determined by immunohistochemistry, from both diabetic and non-diabetic patients.** R^2^: Coefficient of correlation; (IOD: Integrated optical density).

Human monocytes derived macrophages (HMDM) were obtained from all twenty patients and used for the determination of CRP content by immunohistochemistry as well as for determination of CRP mRNA by real time PCR. CRP content as well as CRP mRNA expression in HMDM from diabetic patients were slightly higher in comparison to non-diabetic patients; however these increments were statistically non-significant (Table 2). Interleukin 6 levels in HMDM were analyzed by immunohistochemistry and no significant differences were observed between diabetic patients and non-diabetic patients (Table 2).Carotid lesions were analyzed for their CRP content by immunohistochemistry. CRP content in carotid lesions was significantly higher by 62% in diabetic patients in comparison to the non-diabetic patients (p = 0.05), thus indicating that atherosclerotic lesion from diabetic patients were characterized by higher CRP levels and higher levels of inflammation (Figure 1A). Similarly, IL-6 content in carotid lesions was significantly higher by 74% in diabetic patients in comparison to non-diabetic patients (Figure 1B).CRP levels in carotid plaques were probably locally secreted by arterial macrophages since CRP staining in the carotid lesions colocalized with CD68 staining, which represents macrophage cells (Figure 2).When plotting CRP content in carotid artery from all twenty patients enrolled in this study, against CRP content in HMDM, a significant positive correlation was observed (r2 = 0.611, Figure 3). Similarly, when plotting CRP content in carotid artery from all the twenty patients enrolled in this study, against CRP mRNA expression in HMDM, a significant positive correlation was also observed (r2 = 0.661, Figure 4). These results illustrate that CRP content and expression in HMDM could reflect CRP content in carotid plaques, in both diabetic as well as non diabetic patients. Interestingly when plotting CRP content in carotid artery from all the twenty patients enrolled in this study, against systemic CRP serum levels, no significant correlation could be obtained (data not shown), thus further proving that systemic CRP is not a reliable marker for evaluating carotid plaque CRP levels.

**Table 2:** Human Monocytes derived macrophages (HMDM) isolated from diabetic versus non diabetic patients were analyzed for their C-Reactive Protein (CRP) and Interleukin-6 (IL-6) content by immunohistochemistry as well as for their CRP mRNA levels by real time PCR

	**Diabetic patients (n = 10)**	**Non-diabetic patients (n = 10)**	**P value**
CRP Content in HMDM (IOD)	120401 ± 44479	107442 ± 40119	N.S.
HMDM CRP mRNA/β-Actin mRNA (Arbitrary Unit)	0.94 ± 0.33	0.74 ± 0.37	N.S.
IL-6 Content in HMDM (IOD)	34254 ± 23354	35302 ± 28979	N.S.

CRP and IL-6 content in HMDM from diabetic versus non-diabetic patients.

(N.S.: non-significant; IOD: integrated optical density).

## Discussion

The patients enrolled in this study were divided into two groups: diabetic and non diabetic patients. The diabetic patients were characterized by significantly higher levels of fasting serum glucose as well as of hemoglobin A1C. Serum lipid profile, concomitant diseases and drug therapy (besides hypoglycemic therapy) were similar in both group of patients, therefore changes in carotid lesion from diabetic patients could be related to additional factors beside hypercholesterolemia, hypertension or differences in drug therapy.

CRP content as well as IL-6 levels were significant higher in carotid lesions from diabetic patients in comparison to non-diabetic patients. These results clearly illustrate the higher inflammatory content of atherosclerotic lesions from diabetic patients. These results are compatible with previous studies showing higher inflammatory characteristics in vascular cells and in coronary plaques from diabetic patients [23,24], illustrated by higher levels of cytokines, TNFα, adhesion molecules and chemo attractant factors release in diabetic patients in comparison to non-diabetic patients.

CRP content in the carotid lesion could be produced by arterial macrophages since CRP staining in the lesions co-localized with CD68, a specific staining for macrophages as previously shown [15,25]. CRP locally produced could be responsible for the higher levels of IL-6 measured in carotid lesions from diabetic patients or inversely, since CRP was both shown to be regulated by dual action of IL1 and IL6 and shown to induce IL-6 synthesis and release from cells of the atherosclerotic lesion [26].

These results are compatible with previous studies showing both CRP mRNA and protein localized in atherosclerotic lesions, predominantly in vascular smooth muscle cells and macrophages [27]. Supporting this hypothesis, inflammatory stimuli such as TNFα or combination of IL1/IL6 have been found to induce CRP-production from human coronary artery cells [28].

CRP content and mRNA expression as well as IL6 content were determined in human monocytes derived macrophages. No significant differences in these parameters could be detected between diabetic and non diabetic patients. However, when plotting CRP carotid artery content against CRP HMDM content or against CRP mRNA expression in HMDM, a significant positive correlation was obtained. These results illustrate that since HMDM and arterial macrophages differentiate from a common monocytic cellular lineage, HMDM could reflect and undergo similar regulation to that observed in arterial lesion. It has been previously shown that monocytes will differentiate into specific macrophage subpopulations (similar to that located in tissues) in response to alternative cytokine environments [29]. Moreover, circulating monocytes of patients with cardiovascular disease have been shown to display high levels of surface receptors that may be involved in inflammatory responses [30]. If indeed carotid plaques CRP correlates with HMDM CRP, providing that atherosclerotic lesion cannot be easily obtained for diagnostic purposes, HMDM obtained from blood samples could be easily available, to assess atherosclerotic lesion inflammatory status.

CRP produced by macrophages, unlike systemic hepatocyte-derived CRP, could be selectively up-regulated following pro-atherosclerotic factors. Although we expect macrophage CRP levels to be significantly lower than systemic hepatocyte-derived CRP, these levels are expected to be specific to atherosclerotic development and not influenced by general systemic inflammatory processes that usually lead to massive increase in serum CRP, a positive acute phase reactant. This was further illustrated by the highly heterogeneous blood CRP levels in diabetic patients observed in this study.

These results could also shed some light on the controversy of whether serum CRP is indeed a biomarker for cardiovascular diseases and whether it is a pro-atherogenic factor [31,32]. If, CRP is produced from different sources [26] (including blood monocytes derived macrophages) and if as we hypothesize, CRP regulation is cell specific, this could lead to an heterogeneous population of CRP molecules. CRP locally produced in the atherosclerotic lesion could be structurally different from circulating CRP, including dissociation into monomers instead of the pentameric structure of hepatocyte-produced CRP [33-35].Therefore by referring only to systemic CRP, we are losing precious information on CRP from additional sources and this could explain discrepancies between studies on CRP casual involvement in cardiovascular diseases.

We conclude that pro-inflammatory effects mediated by CRP in the arterial wall could be caused by locally secreted macrophage CRP and that measurement of CRP in blood-derived macrophages could reflect atherosclerotic macrophages secretion of CRP. Further studies are needed however to investigate the correlation between levels of CRP produced by arterial macrophages, and the prediction of coronary artery disease. Understanding of locally produced macrophage CRP in the arterial wall during atherogenesis could be of major importance in our understanding of the mechanisms underlying inflammatory response pathways during atherogenesis.

## Competing interests

The authors declare that they have no competing interests.

## Authors’ contributions

MK mainly designed the study, performed the analysis and interpretation of the data and drafted the manuscript.SH was involved in the selection of the patients, preparation of the helsinky approval and acquisition of the clinical data. YT was mainly involved with the lesion tissues preparation for immunohistochemistry and acquisition of immunohistochemistry analyses. EM and AL were mainly involved in the PCR experiments and monocytes derived macrophages isolation. SN was involved in the acquisition of the data from the carotid lesion and interpretation of the data. TH was involved in the design of the study and draft of the manuscript. All authors read and approved the final manuscript.
